# Supplementary material for: Gas network value chain optimization considering compressor capacity constraints
Source: PLoS One. 2026 Mar 30;21(3):e0332833. doi: 10.1371/journal.pone.0332833 (PMC13035171; doi:10.1371/journal.pone.0332833)
Supplement: Attachment — Submitted filename: Response to Reviewers.docx [file pone.0332833.s002.docx]

First and foremost, we want to thank the editor and the reviewer for your careful review and constructive comments of our manuscript. We have read the comments carefully and revised our paper accordingly. Our point-to-point responses to the comments are provided below. For the ease of reading, all of our responses are in blue font color, and are started with [Authors]. Any inserted, replaced or modified texts to the manuscript are in italics in this document. In addition, we use the red-colored font to highlight some important changes/modifications in the revised manuscript.

**Reviewer #1:**

1. The paper lacks in major contribution and motivation of research.

[Authors] Thanks for the comment. In the revised manuscript, we have thoroughly reorganized and rewritten the Introduction section to better highlight the motivation behind our work and to clearly position it within the context of existing research. Furthermore, we have added a new Section 2.6 titled "Our Contributions", where we explicitly summarize and clarify the major contributions of this study.

2. The background and significance of this study should be highlighted in the abstract.

[Authors] Thanks for the comment. We have carefully reorganized and rewritten the Abstract section to better emphasize the background and significance of our research. The revised abstract now provides a clearer context of the problem being addressed and highlights the importance of our study in the field.

3. Check the English presentation of this paper to remove the typo mistakes. Some grammatical issues need to be addressed in the whole text. Please reform the long paragraphs. Please polish the writing and English of the manuscript carefully. The writing of the paper needs a lot of improvement in terms of grammar, spelling, and presentation. The paper needs careful English polishing since there are many typos and poorly written sentences. I found several errors.

[Authors] Thank you for pointing this out. We have carefully reviewed the entire manuscript and made comprehensive revisions to improve the English presentation. This includes correcting grammatical errors, eliminating typos, restructuring overly long paragraphs, and enhancing the overall clarity and readability of the text. We have polished the writing throughout the manuscript to ensure it meets the standards of academic English.

4. In the "Introduction" section, a more detailed analysis of the existing literature on the subject is needed, and an in-depth analysis of the possible application fields.

[Authors] Thanks for the comment. We have expanded the literature review and conducted a more detailed analysis of the existing studies in Section 2: Literature Review. This revision not only provides a clearer overview of the current research landscape but also discusses the potential application fields of our study in greater depth.

5. The mathematics used throughout the article is still not very strict. Please try to update and illustrate some elements in the mathematical model that are not defined very strictly.

[Authors] Thanks for the comment. In the revised version of the manuscript, we have carefully reviewed the mathematical formulations and made the necessary modifications to ensure greater rigor and clarity. Specifically, we have refined the definitions of key elements and improved the consistency and accuracy of the notations used in our model.

6. Please categorize your and previous research in the Table in the section
Literature Review to show the better research gap.

[Authors] Thanks for the comment. Thank you for your suggestion. While we did not add a table, we have revised Section 2: Literature Review to include a more detailed comparative analysis between previous studies and our own research. This enhanced discussion more clearly outlines the differences in methodology, focus, and contributions, thereby better highlighting the research gap that our work addresses.

7. The overall structure of the article should be improved.

[Authors] Thank you for your suggestion. In the revised manuscript, we have reorganized the overall structure of the paper to improve its clarity and logical flow. The updated structure now consists of seven sections: Introduction, Literature Review, Problem Description, The Mathematical Programming Model, Solution Algorithm, Numerical Experiment, Conclusions and Future Works.

8. The result part is week, results and discussion should be better explained.

[Authors] Thanks for the comment. In the revised manuscript, we have enriched the Numerical Experiment section by providing more detailed explanations of the experimental results and further elaborating on their implications. We also enhanced the discussion to better interpret the findings, compare them with existing methods, and highlight the strengths and limitations of our approach.

9. References must be updated.

[Authors] Thanks for the comment. We have updated the reference list by adding and incorporating more recent and relevant literature to reflect the current state of research in this field.

10. Check all of your Figures and Tables have a good explanation of your text.

[Authors] Thanks for the comment. We have carefully checked and revised all the figures and tables in the manuscript to ensure that each one is clearly explained and well-integrated into the main text. We also revised the corresponding captions and descriptions to improve clarity and consistency, making it easier for readers to understand their relevance and role in supporting our research findings.

11. Many paragraphs without citations.

[Authors] Thank you for pointing this out. We have thoroughly reviewed the manuscript and revised the relevant sections to ensure that appropriate citations are provided wherever necessary.

12. What are the contributions and novelty of work mentioned?

[Authors] Thanks for the comment. To clearly present the contributions and novelty of our work, we have added a new Section 2.7 titled "Our Contributions" in the revised manuscript. In this section, we explicitly summarize the key innovations and contributions of our research.

13. The authors' conclusions need to be improved, a comparison of the results obtained with those already existing in the literature would be appropriate. I suggest also describing what can still be improved in this work, which can still be improved based on the results obtained, according to the authors' view. It is suggested to offer some limitations existed in this study and an outlook for future study in the last section.

[Authors] Thanks for the comment. In the revised manuscript, we have enhanced the *Conclusions and Future Works* section by providing a more comprehensive summary of our key findings and their implications. Furthermore, we have added a critical discussion of the limitations of the current study and outlined several potential directions for future research based on the observed results.

**Reviewer #2: General Comment:**
The manuscript presents a promising study; however, several areas need improvement to enhance its clarity, readability, and scientific contribution. The authors should address issues related to language quality, novelty presentation, and proper referencing. Additionally, improving the figures, expanding the literature review, and providing more detail in the conclusions section would strengthen the manuscript significantly. Below are more specific comments to guide the authors in revising the paper.

[Authors] We sincerely thank the reviewer for the positive recognition of the potential value of our study, as well as for the detailed and constructive feedback. We will address your suggestions as follows:

1. Language Quality:
o The manuscript would benefit from a thorough review of its English language usage to improve clarity and readability. It is recommended that the authors address grammatical and syntactic errors throughout the text to enhance the quality of the writing.
[Authors] Thanks for the comment. We have carefully reviewed and revised the entire manuscript to correct grammatical, syntactic, and typographical errors. These revisions were made to improve the overall clarity, readability, and quality of the writing.

2. Novelty and Original Contribution:
o The novelty of the research is not clearly highlighted. The authors should explicitly articulate the unique contributions of their work to distinguish it from existing studies.
[Authors] Thanks for the comment. We have added a new section (Section 2.6: Our Contributions) in the revised manuscript. This section clearly summarizes the key innovations and original contributions of our research in comparison to existing methods.

3. Nomenclature Section:
o To improve the manuscript's readability, the authors are encouraged to include a nomenclature section at the beginning. This should contain all variables, acronyms, indices, and constants used in the manuscript, allowing readers to refer easily to these definitions.
[Authors] Thank you for the suggestion. In the revised manuscript, we have added a list of acronyms and abbreviations at the beginning to enhance readability. Additionally, we have provided detailed definitions and classifications of all key variables, parameters, and indices in Tables 1 to 3.

4. Introduction and Literature Review:
o The Introduction would benefit from a more thorough review of relevant literature. A comprehensive manuscript in this domain typically references a minimum of 40 studies. Expanding the literature review will also provide a stronger foundation for the study's relevance and innovation.

[Authors] Thanks for the comment. In the revised manuscript, we have expanded Section 2: Literature Review to include a broader and more comprehensive survey of relevant studies in this field. This enhancement helps to better position our work within the existing body of literature and to strengthen the foundation for the research’s relevance and innovation. The number of references has also been increased accordingly to reflect the depth of the revised review.

5. Recommended References:
o The following references are highly relevant to the subject and should be incorporated to enhance the background and context of the study:
♣ Detection of internal fault in differential transformer protection based on fuzzy method. International Journal of Physical Sciences, 6(26), pp.6150-6158, 2011.
♣ Distributed generation management in smart grid with the participation of electric vehicles with respect to the vehicle owners’ opinion by using the imperialist competitive algorithm. Sustainability, 14(8), p.4770, 2022.
♣ Optimum operation management of microgrids with cost and environment pollution reduction approach considering uncertainty using multi‐objective NSGAII algorithm. IET Renewable Power Generation, 2022.
♣ Economic dispatch optimization considering operation cost and environmental constraints using the HBMO method. Energy Reports, 10, pp.1718-1725, 2023.
♣ Unit commitment for power generation systems based on prices in smart grid environment considering uncertainty. Sustainability, 13(18), p.10219, 2021.
♣ Locating and sizing of capacitor banks and multiple DGs in distribution system to improve reliability indexes and reduce loss using ABC algorithm. Bulletin of Electrical Engineering and Informatics, 10(2), pp.559-568, 2021.
♣ Comparison of SVC and STATCOM in static voltage stability margin enhancement. International Journal of Electrical and Computer Engineering, 3(2), pp.297-302, 2009.
♣ Optimal management of energy storage systems for peak shaving in a smart grid. Computers, Materials and Continua, 75(2), pp.3317-3337, 2023.
♣ Optimal design of solar–wind hybrid system-connected to the network with cost-saving approach and improved network reliability index. SN Applied Sciences, 1(12), p.1742, 2019.
[Authors] Thanks for the comment. All of the suggested references have been carefully reviewed and incorporated into the Literature Review section of the revised manuscript, as they provide valuable background and context for our study.

6. Figures Quality:
o Figures in the manuscript should be provided in higher resolution to improve clarity. Each figure should also be thoroughly described within the text to ensure that it contributes effectively to the manuscript's presentation.
[Authors] Thanks for the comment. In the revised manuscript, we have replaced the figures with higher-resolution versions to improve clarity and ensure that they meet the quality standards. Additionally, we have provided more detailed descriptions of each figure within the text to ensure that they effectively contribute to the manuscript's presentation and enhance the understanding of the results.

7. Future Work and Limitations:
o A brief discussion of potential limitations and directions for future research should be included in the Conclusions and Recommendations section. This will provide a more comprehensive view of the study’s scope and areas for future exploration.

[Authors] Thanks for the comment. In response, we have expanded the Conclusions and Future Works section to include a brief discussion of the limitations of our current study. We have also outlined several potential directions for future research based on the findings of this work.
