# Supplementary material for: Gas network value chain optimization considering compressor capacity constraints
Source: PLoS One. 2026 Mar 30;21(3):e0332833. doi: 10.1371/journal.pone.0332833 (PMC13035171; doi:10.1371/journal.pone.0332833)
Supplement: Attachment — Submitted filename: response to reviewer 20250903.docx [file pone.0332833.s003.docx]

**Response to Reviewers**

**Manuscript ID:** PONE-D-25-04544R1
**Title:** Gas network value chain optimization considering compressor capacity constraints

Dear Academic Editor and Reviewers,

We sincerely thank the Academic Editor and all reviewers for their constructive comments and valuable suggestions, which have significantly helped us improve the quality and clarity of our manuscript. We have carefully revised the manuscript accordingly. Below, we provide a detailed, point-by-point response to each reviewer’s comments. Changes have been incorporated in the revised manuscript and highlighted accordingly.

**Reviewer #1**

**Comment 1:** *The writing of the paper still needs careful English polishing since there are many typos and poorly written sentences. I found several errors.*

**Response:** We appreciate this important comment. The manuscript has undergone thorough English editing and proofreading to improve readability, grammar, and style. Long and complex sentences have been simplified, and typographical errors have been corrected.

**Comment 2:** *The overall structure of the article should be improved.*

**Response:** We have revised the structure of the manuscript to improve clarity and logical flow. In particular, the **Introduction** has been streamlined to emphasize research motivation and contributions, and the **Literature Review** has been reorganized into thematic subsections.

**Comment 3:** *The result part still week, results and discussion must be better explained.*

**Response:** We reorganized explanatory paragraphs after each major results table, clarifying the accuracy of the SLP approach, its scalability compared to GUROBI, and the impact of compressor capacity constraints.

**Comment 4:** *Add the suitable references from the following list.*

**Response:** We carefully reviewed the suggested references and incorporated several recent and relevant works into **Section 2.6 Emerging Trends in Energy Network Optimization**. For example, we added discussions of studies on microgrid energy management (Abdelsattar et al., 2023; Abdelsattar et al., 2024a), machine learning for renewable energy forecasting (Abdelsattar et al., 2024b; Abdelsattar et al., 2025), and metaheuristic algorithms for hybrid energy systems (Abdelsattar et al., 2024c). These additions strengthen the context of our work and highlight its contribution by addressing compressor capacity constraints in natural gas networks.

**Reviewer #3**

**Comment:** *The responses demonstrate a strong understanding of the reviewers' concerns and a clear plan of action taken in the revised manuscript. The authors frequently indicate specific sections where changes have been made.*

**Response:** We sincerely thank the reviewer for their positive assessment. We have maintained this careful revision strategy in the current version and ensured that all changes are clearly indicated in the revised manuscript.

**Reviewer #4**

**Comment 1:** *The manuscript suffers from several critical formatting errors and areas where clarity could be improved.*

**Response:** We have corrected all formatting issues. In particular, broken citations previously appearing as “Error! Reference source not found.” have been fixed, and consistency in headings, figures, and tables has been ensured.

**Comment 2:** *There is a significant contradiction regarding data availability. The submission form states GitHub availability, but the text says “contact the author.” This inconsistency must be resolved.*

**Response:** We thank the reviewer for pointing out this contradiction. We have revised the **Data Availability** statement to unify the description. All data are now clearly stated as being openly available on GitHub:
[https://github.com/masonYau/gasNetworkData/tree/main](https://github.com/masonYau/gasNetworkData/tree/main?utm_source=chatgpt.com)

**Comment 3:** *Some sentences could be more concise.*

**Response:** We have simplified several overly complex sentences throughout the manuscript. For example, the sentence about compressor compression ratio was revised to:

“A compressor’s compression ratio is a complex, nonlinear function of its operating conditions, including inlet flow rate and rotational speed.”
This makes the text clearer and more concise.

**Final Remarks**

We believe that the revised manuscript has significantly improved in terms of language, structure, results interpretation, and consistency. We thank the reviewers again for their helpful feedback and look forward to your favorable consideration.
